# Supplementary material for: Aβ25-35-induced autophagy and apoptosis are prevented by the CRMP2-derived peptide ST2-104 (R9-CBD3) via a CaMKKβ/AMPK/mTOR signaling hub
Source: PLoS One. 2024 Sep 26;19(9):e0309794. doi: 10.1371/journal.pone.0309794 (PMC11426444; doi:10.1371/journal.pone.0309794)

FIGURE 2

Group：Con、$A\beta_{25-35}$、ST2-104、$A\beta_{25-35}$+ST2-104

identity of experimental samples：cell protein

BAX 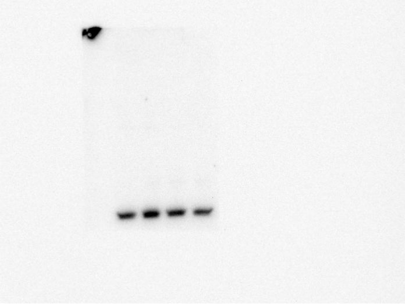 BAX-ACTIN

BCL-2 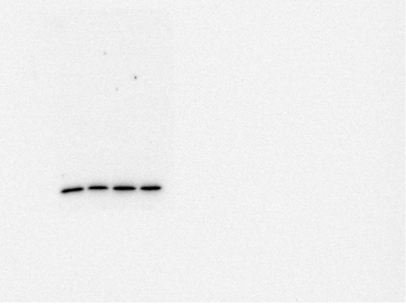 BCL-2-ACTIN 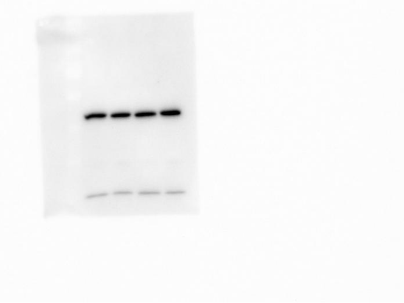

CC3 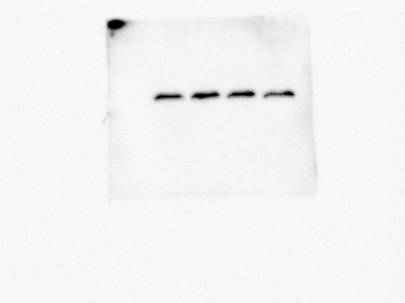 C3 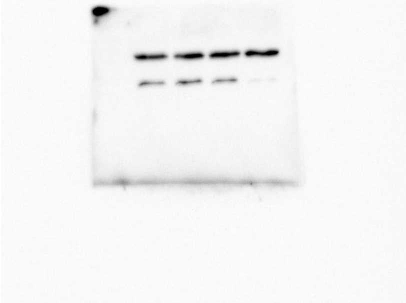

FIGURE 3

Group：Con、$A\beta_{25-35}$、ST2-104、$A\beta_{25-35}$+ST2-104

identity of experimental samples：cell protein

BECLIN-1 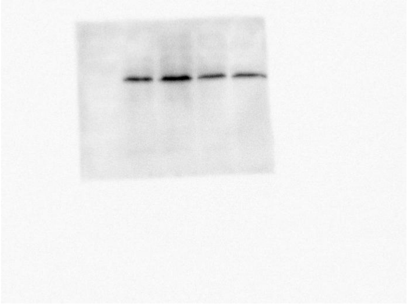 LC3 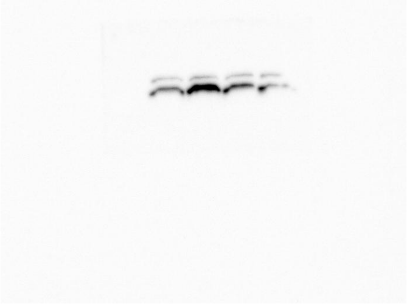

FIGURE 4

Group：Con、$A\beta_{25-35}$、RAPA、$A\beta_{25-35}$+ST2-104、$A\beta_{25-35}$+ST2-104+RAPA

identity of experimental samples：cell protein

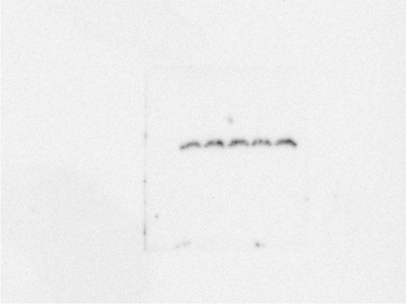

BAX

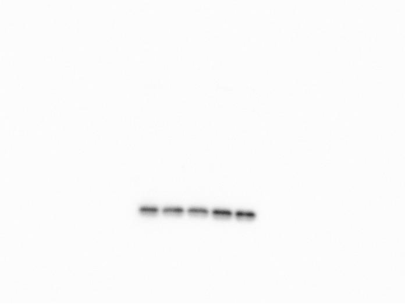

BCL-2

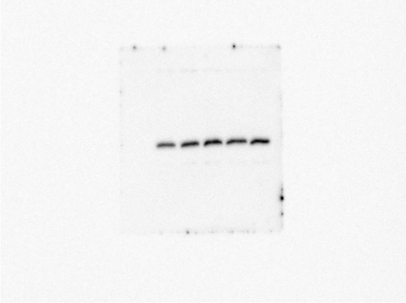

ACTIN

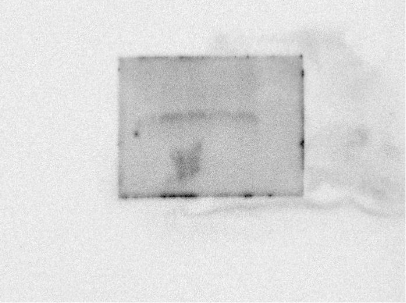

CC3

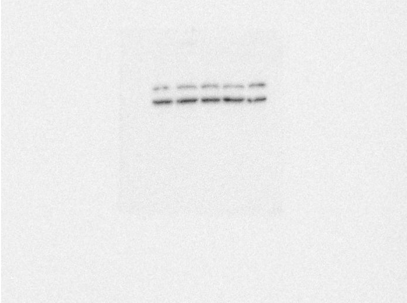

C3

FIGURE 6

Group：Con、$A\beta_{25-35}$、ST2-104、$A\beta_{25-35}$+ST2-104

identity of experimental samples：cell protein

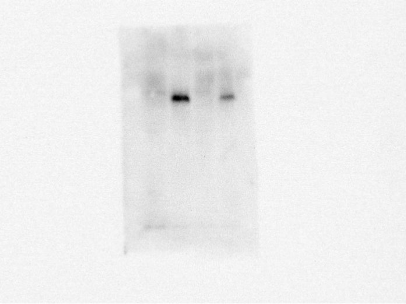

P-AMPK

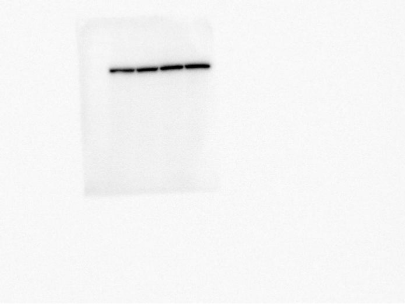

AMPK

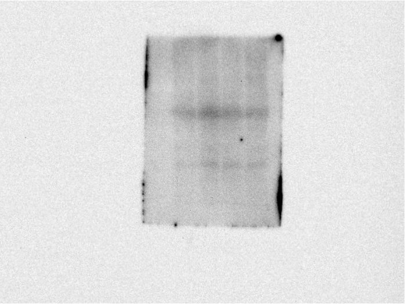

CAMKK

FIGURE 7

Group: Con、$A\beta_{25-35}$、$A\beta_{25-35}$+ST2-104、$A\beta_{25-35}$+STO-609、$A\beta_{25-35}$+ST2-104+STO-609

identity of experimental samples： cell protein

BAX

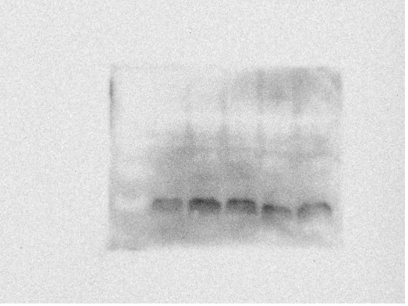

BCL-2

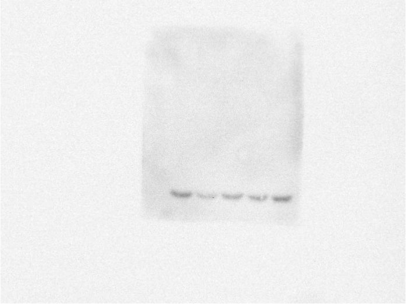

ACTIN

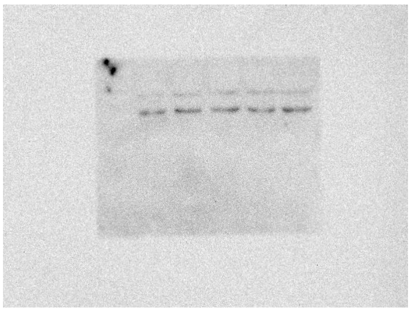

CC3

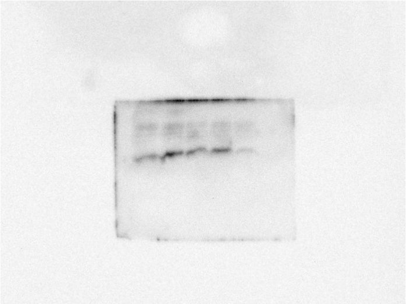

C3

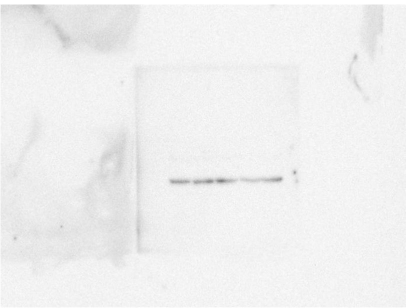

FIGURE 8

Group: Con、$A\beta_{25-35}$、$A\beta_{25-35}$+ST2-104、$A\beta_{25-35}$+STO-609、$A\beta_{25-35}$+ST2-104+STO-609

identity of experimental samples： cell protein

BECLIN-1 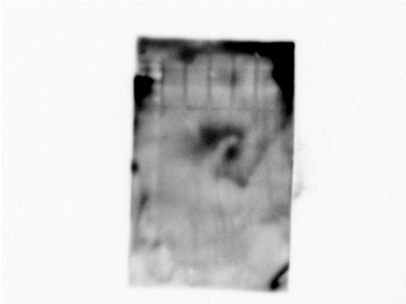

LC3 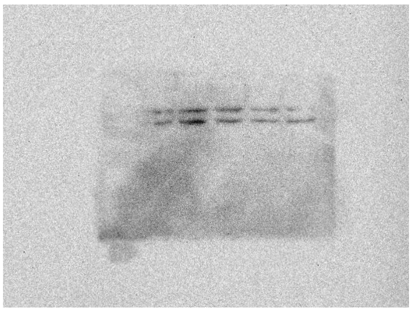

Supplement: S1 Raw images — (PDF) [file pone.0309794.s001.pdf]
