# Supplementary material for: Aβ25-35-induced autophagy and apoptosis are prevented by the CRMP2-derived peptide ST2-104 (R9-CBD3) via a CaMKKβ/AMPK/mTOR signaling hub
Source: PLoS One. 2024 Sep 26;19(9):e0309794. doi: 10.1371/journal.pone.0309794 (PMC11426444; doi:10.1371/journal.pone.0309794)
Supplement: Attachment — Submitted filename: Response to Reviewers.docx [file pone.0309794.s002.docx]

**PONE-D-23-36719**

**Response to reviewers**

Our responses are indicated in red font below.

Journal requirements:

When submitting your revision, we need you to address these additional requirements.

1. Please ensure that your manuscript meets PLOS ONE's style requirements, including those for file naming. The PLOS ONE style templates can be found at
https://journals.plos.org/plosone/s/file?id=wjVg/PLOSOne_formatting_sample_main_body.pdf and
<https://journals.plos.org/plosone/s/file?id=ba62/PLOSOne_formatting_sample_title_authors_affiliations.pdf>

2. Thank you for stating the following financial disclosure: 
 [This study was supported by the grant provided by the Major Chronic Disease Program of the Ministry of Science and Technology of China (No. 2018YFC1312301), the General Program of the National Natural Science Foundation of China (No. 82071442), the Jilin Provincial Department of Finance（JLSWSRCZX2021-004）.].  
Please state what role the funders took in the study.  If the funders had no role, please state: "The funders had no role in study design, data collection and analysis, decision to publish, or preparation of the manuscript." 
If this statement is not correct you must amend it as needed. 
Please include this amended Role of Funder statement in your cover letter; we will change the online submission form on your behalf. We have added this to the cover letter.

3. We note that your Data Availability Statement is currently as follows: [All relevant data are within the manuscript and its Supporting Information files]

Please confirm at this time whether or not your submission contains all raw data required to replicate the results of your study. Authors must share the “minimal data set” for their submission. PLOS defines the minimal data set to consist of the data required to replicate all study findings reported in the article, as well as related metadata and methods (https://journals.plos.org/plosone/s/data-availability#loc-minimal-data-set-definition).

For example, authors should submit the following data:

- The values behind the means, standard deviations and other measures reported;
- The values used to build graphs;
- The points extracted from images for analysis.

Authors do not need to submit their entire data set if only a portion of the data was used in the reported study.

If your submission does not contain these data, please either upload them as Supporting Information files or deposit them to a stable, public repository and provide us with the relevant URLs, DOIs, or accession numbers. For a list of recommended repositories, please see https://journals.plos.org/plosone/s/recommended-repositories.

If there are ethical or legal restrictions on sharing a de-identified data set, please explain them in detail (e.g., data contain potentially sensitive information, data are owned by a third-party organization, etc.) and who has imposed them (e.g., an ethics committee). Please also provide contact information for a data access committee, ethics committee, or other institutional body to which data requests may be sent. If data are owned by a third party, please indicate how others may request data access.

We have provided all the raw data in an accompanying Supplementary file.

**Additional Editor Comments:**

Fig 1D, as the concentration of ST2-104 increased from 5 uM to 40 uM, the protective effects was not increasing. Instead, it seemed decreasing. The authors should discuss why.

While the exact reason for why we did not observe increasing neuroprotection upon increasing the concentration of ST2-104 is not known, we hypothesize that this may be due to stoichiometric saturation at the lowest concentration of 5 µM.

Fig 3. Autophagy flux should be examined with a liposome inhibitor such as chloroquine. We indeed plan to measure this in future work.

Fig 7 and Fig 8 lacking a STO-609 control. Controls for STO-609 have been previously published (see PMIDs: 37710272 and 36889111). While we should have used it as a control in our studies, we acknowledge this as an oversight and limitation of our work here.


Reviewers' comments:

Reviewer's Responses to Questions

**Comments to the Author**

1. Is the manuscript technically sound, and do the data support the conclusions?

The manuscript must describe a technically sound piece of scientific research with data that supports the conclusions. Experiments must have been conducted rigorously, with appropriate controls, replication, and sample sizes. The conclusions must be drawn appropriately based on the data presented.

Reviewer #1: Yes

2. Has the statistical analysis been performed appropriately and rigorously?

Reviewer #1: Yes

3. Have the authors made all data underlying the findings in their manuscript fully available?

The [PLOS Data policy](http://www.plosone.org/static/policies.action#sharing) requires authors to make all data underlying the findings described in their manuscript fully available without restriction, with rare exception (please refer to the Data Availability Statement in the manuscript PDF file). The data should be provided as part of the manuscript or its supporting information, or deposited to a public repository. For example, in addition to summary statistics, the data points behind means, medians and variance measures should be available. If there are restrictions on publicly sharing data—e.g. participant privacy or use of data from a third party—those must be specified.

Reviewer #1: No

4. Is the manuscript presented in an intelligible fashion and written in standard English?

PLOS ONE does not copyedit accepted manuscripts, so the language in submitted articles must be clear, correct, and unambiguous. Any typographical or grammatical errors should be corrected at revision, so please note any specific errors here.

Reviewer #1: Yes

5. Review Comments to the Author

Please use the space provided to explain your answers to the questions above. You may also include additional comments for the author, including concerns about dual publication, research ethics, or publication ethics. (Please upload your review as an attachment if it exceeds 20,000 characters)

Reviewer #1: The authors have evaluated the protective mechanism of ST2-104 in Aβ25-35 peptide induced cytotoxicity in SHY-SY5Y cells. They showed that ST2-104 prevented the neuronal apoptosis by inhibiting autophagy through a CaMKKβ/AMPK/mTOR signaling pathway. Study of the mechanism of Aβ neurotoxicity is invaluable. Overall, there are several concerns regarding the methodology and interpretation of results.


Major Comments:
Title: Aβ25-35-induced autophagy and apoptosis are inhibited by the CRMP2-derived peptide ST2-104 (R9-CBD3) via a CaMKKβ/AMPK/mTOR signaling hub
Comment: The title should accurately describe the contents of the manuscript. Since the authors used the ST2-104 peptide as a pre-treatment, Aβ25-35-induced autophagy and apoptosis are prevented not inhibited .............
We have amended the title to reflect the prevention.

Comments (Methodology and results):
Line 133 &139: What was the solvent of Aβ25-35? DMSO or acetic acid?
Control group received no vehicle? These should be addressed in the method section.
The solvent for Aβ_25-35_ was acetic acid, and an equal volume of acetic acid was added to the control group. we will modify the content in the Methods section.

In Line 182, the authors mentioned the use of One-way analysis of variance. The authors should provide the F values in the result section. F-values for the relevant data are provide in the Supplementary excel file.

The authors have stated in line 280, the percentage of cells in gate (M1). They should provide the flow cytometry protocol of gating and quantification, background noise detection, and an unstained sample. We provide examples of the gating, quantification, noise detection and an unstained sample in a supplementary figure.

Comments (Discussion):
In the line 349-356 of Discussion,: The sentences are suitable for introduction not discussion. Noted and removed.

The SH-SY5Y cells have two phenotypes “N” and “S” types (Encinas et al. J Neurochem 75:991–1003, 2000). These should be discussed in relation to the current results. We now state that “The SH-SY5Y cell line has been described to produce both substrate adherent (S-type) and neuroblastic (N-type) cells that can undergo transdifferentiation. Therefore, even though the SH-SY5Y cell line is derived from triple successive subclone selection of N-type cells, it contains a small proportion of S-type cells.”

Please see the references:

Forster JI, Köglsberger S, Trefois C, Boyd O, Baumuratov AS, Buck L, Balling R, Antony PM. Characterization of Differentiated SH-SY5Y as Neuronal Screening Model Reveals Increased Oxidative Vulnerability. J Biomol Screen. 2016 Jun;21(5):496-509. doi: 10.1177/1087057115625190. Epub 2016 Jan 6. PMID: 26738520; PMCID: PMC4904349.

In Line 389, the authors stated that “our data supports an anti-autophagic role for ST2-104 in the face of Aβ25-35 induced autophagy. Since the authors performed the pre-treatment, it is true to write ST2-104 prevented Aβ25-35 induced autophagy. We have corrected this.

Please omit “we” in Line 399: "We found that when we autophagy was induced with RAPA, the nuclear condensation and the expression of Bax, C-caspase-" Done.

6. PLOS authors have the option to publish the peer review history of their article ([what does this mean?](https://journals.plos.org/plosone/s/editorial-and-peer-review-process#loc-peer-review-history)). If published, this will include your full peer review and any attached files.


If you choose “no”, your identity will remain anonymous but your review may still be made public.


**Do you want your identity to be public for this peer review?** For information about this choice, including consent withdrawal, please see our [Privacy Policy](https://www.plos.org/privacy-policy).

Reviewer #1: No

[NOTE: If reviewer comments were submitted as an attachment file, they will be attached to this email and accessible via the submission site. Please log into your account, locate the manuscript record, and check for the action link "View Attachments". If this link does not appear, there are no attachment files.]

While revising your submission, please upload your figure files to the Preflight Analysis and Conversion Engine (PACE) digital diagnostic tool, <https://pacev2.apexcovantage.com/>. PACE helps ensure that figures meet PLOS requirements. To use PACE, you must first register as a user. Registration is free. Then, login and navigate to the UPLOAD tab, where you will find detailed instructions on how to use the tool. If you encounter any issues or have any questions when using PACE, please email PLOS at <a href="mailto:figures@plos.org">figures@plos.org. Please note that Supporting Information files do not need this step.
